# An eHealth Capabilities Framework for Graduates and Health Professionals: Mixed-Methods Study

**DOI:** 10.2196/10229

**Published:** 2018-05-15

**Authors:** Melissa Brunner, Deborah McGregor, Melanie Keep, Anna Janssen, Heiko Spallek, Deleana Quinn, Aaron Jones, Emma Tseris, Wilson Yeung, Leanne Togher, Annette Solman, Tim Shaw

**Affiliations:** ^1^ Research in Implementation Science and eHealth Faculty of Health Sciences The University of Sydney Camperdown Australia; ^2^ Speech Pathology Graduate School of Health The University of Technology Sydney Ultimo Australia; ^3^ Faculty of Health Sciences The University of Sydney Camperdown Australia; ^4^ School of Dentistry The University of Sydney Westmead Australia; ^5^ Sydney Local Health District Camperdown Australia; ^6^ Faculty of Arts and Social Sciences The University of Sydney Camperdown Australia; ^7^ eHealth NSW Chatswood Australia; ^8^ NSW Health Education Training Institute Gladesville Australia

**Keywords:** telemedicine, mobile health, clinical competence, education, professional, education, graduate

## Abstract

**Background:**

The demand for an eHealth-ready and adaptable workforce is placing increasing pressure on universities to deliver eHealth education. At present, eHealth education is largely focused on components of eHealth rather than considering a curriculum-wide approach.

**Objective:**

This study aimed to develop a framework that could be used to guide health curriculum design based on current evidence, and stakeholder perceptions of eHealth capabilities expected of tertiary health graduates.

**Methods:**

A 3-phase, mixed-methods approach incorporated the results of a literature review, focus groups, and a Delphi process to develop a framework of eHealth capability statements.

**Results:**

Participants (N=39) with expertise or experience in eHealth education, practice, or policy provided feedback on the proposed framework, and following the fourth iteration of this process, consensus was achieved. The final framework consisted of 4 higher-level capability statements that describe the learning outcomes expected of university graduates across the domains of (1) digital health technologies, systems, and policies; (2) clinical practice; (3) data analysis and knowledge creation; and (4) technology implementation and codesign. Across the capability statements are 40 performance cues that provide examples of how these capabilities might be demonstrated.

**Conclusions:**

The results of this study inform a cross-faculty eHealth curriculum that aligns with workforce expectations. There is a need for educational curriculum to reinforce existing eHealth capabilities, adapt existing capabilities to make them transferable to novel eHealth contexts, and introduce new learning opportunities for interactions with technologies within education and practice encounters. As such, the capability framework developed may assist in the application of eHealth by emerging and existing health care professionals. Future research needs to explore the potential for integration of findings into workforce development programs.

## Introduction

### Background

Developing an eHealth-ready workforce [1,2] is becoming a key priority for addressing the complex challenges in health care globally [3,4]. Modern health care now incorporates a multitude of eHealth technologies that can be used for (1) monitoring, tracking, and informing health; (2) interacting for health such as using digital technologies to enable health communication among practitioners and between health professionals and clients or patients; and (3) data that enable health via collecting, managing, and using health data to improve outcomes [5]. eHealth has been recognized as pivotal in recent health reforms, with the potential to provide more efficient, cost-effective care with better outcomes [4,6,7]. As such, health services increasingly expect health professionals, new graduates, and experienced workforce alike, to be eHealth ready within practice across diverse digital health environments [4,8]. In turn, this is directing efforts to integrate eHealth education into clinical health degrees, specifically allied health, nursing, pharmacy, dentistry, and medical programs [1,2,9].

Significant work has been conducted by Gray et al (2014) to identify the educational needs of health professionals necessary for an eHealth-capable workforce [9]. A key factor limiting the eHealth readiness of current and future health professionals is the lack of coordinated, formal education in the use of digital technologies in health [9]. The health practice context has become vastly more complex, mirroring changes in patients, the health system, and medical science, making it increasingly challenging for clinicians to safely and efficiently navigate health care [10]. As digital health becomes more widespread, so does the requirement for health professionals to be well versed in navigating and using such technologies [3]. eHealth education, therefore, needs to be coordinated to explain and explore eHealth in both current and future health care contexts, and to incorporate clear specifications as to the eHealth capabilities expected of the current and future health care workforce [9]. Research into, and the development of, coordinated approaches to eHealth curriculum design and implementation are necessary to effectively integrate eHealth capabilities among health graduates and to support the health care workforce in using eHealth technologies [3]. Consequently, specific eHealth education is required, even if students report to be competent and confident in using technology [11]. Such skills must not to be equated with technology proficiency, and most certainly not with information fluency [12]. Information literacy is defined in essence as “the ability to access, evaluate, and use information from a variety of sources” [13] and has become a critical skill for the present generation of students, and indeed for the 21st-century citizen [14,15]. For this to be achieved, a systematic approach to curriculum design and collaborative efforts from stakeholders are needed [9,16].

Multiple issues need to be addressed when designing strategies for embedding eHealth into undergraduate curricula [4,9,16]. Drawing from research into teaching evidence-based medicine [17], eHealth would be more effectively taught (ie, demonstrate improved knowledge, skills, and attitudes) when it is integrated into clinical subjects rather than as an adjunct [16]. Education needs to improve awareness and understanding of the purpose of eHealth in practice contexts, as well as training in the use of technologies [4,9]. Competency frameworks in health informatics [18,19] provide valuable reference points in technical and informatics literacy for workforce development. eHealth-enabled health care, however, extends beyond informatics and requires professionals to integrate digital technologies into the health care and management processes [5]. As such, although health informatics competencies provide a strong foundation for the technical requirements of eHealth, the current and future health workforce must also receive education in the use of eHealth in practice to perform a range of professional functions, such as clinical decision making, patient empowerment, promotion of health and wellness, critical reflection and ethical decision making, and enabling new models of care [3,20]. Although such workforce eHealth competency frameworks are emerging for specific professions [21], limited understanding and agreement remain regarding the core eHealth competencies expected of tertiary graduates.

Although the tertiary education of health professionals is traditionally framed around a set of specific competency standards decreed by professional associations that allow measurable behaviors to be observed and assessed [22], competency frameworks are most beneficial when the skills and practice are consistent across contexts, eg, in the assessment of a specific injury or illness [23]. eHealth practice, on the other hand, might look different in different contexts. For example, effective integration of mobile technologies in one context might involve using mobile devices to enhance the measurement and assessment of injury. In another context, clinicians could be using apps to remotely prescribe exercises and monitor patients’ physical activity. The fluidity in how eHealth skills are demonstrated across different contexts and the speed with which eHealth technologies evolve both clash with the rigidity and speed at which competencies and educational frameworks can be developed. As such, this suggests that competency standards may not be the most appropriate framing of eHealth skills and education [5,22,23].

In contrast to competency, capability has been defined as “a holistic concept that describes how an individual or organization applies their ability in a confident manner to problems in new and unfamiliar circumstances as well as in familiar situations” [24]. As such, a capable workforce includes lifelong learners who are able to identify the need for change, adapt to familiar and novel situations and environments, and work collaboratively with other stakeholders to provide and potentially transform care [22,23]. Although capability has been described as being similar to competence, it in fact encompasses competence and extends beyond the technical skills implied by competence to emphasize the components of adaptability to change, lifelong learning, and self-efficacy [23-25]. As such, capability-informed frameworks address wider aspects of professionalism, focusing on supporting continuous development rather than assessment of a skill at a specific point in time [25].

### Objectives

A capabilities approach is, therefore, able to inform and modify competency-based frameworks that better reflect the complexity of real-life environments as is found in the field of using eHealth for health care and wellness [22,25]. With this in mind, this study aimed to develop an eHealth capabilities framework based on current evidence and stakeholder perceptions of eHealth capabilities that are expected of workforce-ready tertiary health graduates. This study formed one component of a larger project, eHealthMap, which aimed to align the University’s approach to eHealth education with the best evidence and national expectations of workforce-ready graduates. A key focus of the study was to ensure alignment between the required capabilities of new graduates with workforce capability requirements. This led to a collaborative design approach between the University and the New South Wales Ministry of Health.

## Methods

### Triphasic Approach

A 3-phase approach was used. This consisted of a literature review in the first phase, followed by qualitative studies to identify relevant themes, gain expert opinions, and raise consensus with key stakeholders in the second stage. In the third phase, data from the 2 previous stages were reviewed using a Delphi process to develop a framework of capability statements for guiding health curricula and measurement of workforce readiness in the use of eHealth technologies for health care. The project occurred between October 2016 and November 2017.

### Literature Review

A systematic search for literature was undertaken during October 2016 to December 2016 to identify papers related to workforce readiness in the use of eHealth technologies. The databases and search engines used included CINAHL, Medline, ERIC, PsychINFO, Google Scholar, and Google. Databases and search engines were systematically searched for literature on eHealth competencies using combinations and variations of the following key search terms: *eHealth*, *digital health*, *health professional*, *workforce readiness*, *graduate*, *student*, *higher education*, *capability statement*, and *competency*. The fields of health, medicine, nursing, public health, allied health, pharmacy, psychology, physiotherapy, occupational therapy, speech language pathology, dentistry, paramedicine, social work, dietetics, nutrition, radiography, audiology, exercise and sports science, optometry, orthoptics, ophthalmology, and podiatry were included. Researchers also reached out to health and academic communities and reviewed Web pages from a number of professional societies and health organizations to identify the existence of other eHealth readiness competencies and capabilities.

The search was limited to papers published after 2000 to maximize relevance to current clinical contexts and papers written in English. Research studies and reports were included if they (1) referred to readiness competencies or capabilities for students, graduates, or workforce; (2) referred to the use of digital technologies in health care; and (3) provided empirical support for the capability statements or framework. Included peer-reviewed and gray literature were then reviewed to identify existing core competencies or capability statements relating to eHealth readiness. Data were extracted by 1 author (author 2), analysis and synthesis [26] conducted independently by 2 authors (authors 1 and 2), with consensus provided by a third author as required (author 3).

### Focus Groups

Focus groups with key informants from the health workforce and higher education were used to ascertain the perceived eHealth capabilities requirements of new graduates [27]. Purposeful sampling recruited 23 participants with significant expertise or vested interests in eHealth education, practice, or policy. Focus groups were conducted during a workshop held in February 2017 at the University of Sydney. Ethical approval was obtained from the University of Sydney Human Research Ethics Committee (Protocol No. 2016/811) before participant recruitment.

The workshop included 2 focus group sessions: (1) a large focus group (n=23, 1 hour and 14 min) and (2) 4 small breakout focus groups (n=23, with 5-6 participants per group, 45 min). A semistructured script encouraged topic exploration and included the following questions: (1) What eHealth competencies do you expect health graduates to be able to demonstrate? and (2) Could you provide some examples of how these competencies are taught or applied in your organization? Focus group discussions were facilitated by the researcher team experienced in qualitative research methods. To stimulate discussions, participants were presented with evidence of core eHealth readiness competencies identified via the literature review. Of the focus group participants, 65% (15/23) were female, and 48% (11/23) were University of Sydney faculty representatives. Faculty representatives encompassed the fields of physiotherapy, speech pathology, psychology, nursing, dentistry, pharmacy, medicine, information technology and engineering, and mathematics and statistics. The balance of the participants (12/23, 52%) included broad representation from health services (9/23, 39%) and state and national government health agencies (3/23, 13%), including senior executives, clinicians, and senior health administrators. Participants also included recent health professional graduates (2/23, 9%) and 1 enrolled student from the University of Sydney. Interviews were transcribed verbatim and thematically analyzed [26] independently by 2 authors (authors 1 and 2). Codes were applied to the text of the transcripts, with themes systematically refined until saturation was achieved.

### Development of an eHealth Capability Framework

The third phase involved identifying core capability statements by using the Delphi method [28] to refine and establish core eHealth capability statements. This was conducted over 4 iterations [29] during 2017, which allowed participants recruited from the focus groups (n=23) and via invitation to a wider group of key stakeholders (n=16) to systematically consider the capability statements as they evolved. Each round consisted of a meeting and follow-up email correspondence, promoting discussion and consensus between participants. A predetermined quorum of participants (n=12, ie, participation quorum of 50% based on the total number of participants recruited during the focus group phase, where N=23) was present for each round required to reach consensus, and each participant provided feedback throughout the process during meetings or via email. At the end of each round, feedback was incorporated into the capability framework and the revised statements presented for deliberation at the next meeting. Incorporation of feedback resulted in improved clarity and limited redundancy where similar capabilities were collapsed or condensed. Iterations continued until such time as consensus among the participants was achieved [29]. Following consensus agreement, endorsement from key academic and industry organizations was sought.

## Results

### Literature Review

After duplicates were removed, the search revealed 92 relevant papers. Papers were excluded if they were opinion pieces, provided no empirical support, or reported no specific details regarding eHealth capabilities. This left a total of 30 papers included in this review. Emergent themes from the literature were identified (by authors 1 and 2) and refined through research team discussion at regular meetings.

The health, activity, and participation issues identified within the literature were initially arranged into the following 6 categories: (1) health information management (n=24); (2) communication (n=19); (3) professionalism (n=24); (4) information systems and technologies (n=25); (5) patient focus (n=13); and (6) health analytics (n=14). Categorization of the 6 core domains as identified in the literature is presented in Table 1.

### Focus Groups

Three overarching themes, each with multiple subthemes, emerged from the thematic analyses: (1) reinforce fundamental clinical capabilities, (2) acknowledge and adapt existing capabilities, and (3) introduce and provide opportunities for new learning. Themes and subthemes are presented in Table 2 and have been reported previously [20].

### Development of the eHealth Capability Framework

At the completion of the fourth round, consensus was achieved. The final capability framework consisted of 4 overarching domains and 40 performance cues (Multimedia Appendix 1). The 4 upper-level eHealth capability statements describe what an eHealth-ready health graduate should be able to demonstrate (Table 3). The nature of these statements is such that more specific examples of what they might look like in different contexts are needed. These specific examples are provided in the form of performance cues in the framework. As these statements reflect expected capabilities among entry-level clinical positions, levels of mastery are not specified. This would be more appropriate at the curriculum level where educators consider how these capabilities might be developed and assessed in their specific disciplinary and educational context. Feedback from the focus groups indicated that, ideally, the eHealth capability framework would be integrated with relevant professional competency-based occupational standards. Throughout the development process, key stakeholders consistently recommended that the framework needed to incorporate the essential components of both safety and effective communication across all the 4 high-level capability statements.

**Table 1:** Categorization of core domains of eHealth capability as identified in the literature review.

Domain	Summary of capabilities included in the domain	References
Health information management	Recording and storing health information in electronic systems, data quality, and information governance	[2,9,30-46]
Communication	Using digital technologies to support interprofessional relationships, consumer-provider relationships, and multiprofessional care coordination	[2,9,30-46]
Professionalism	Critical appraisal, evidence-based practice, eHealth literacy, continued professional development, ethical use of information, and management and leadership	[2,9,30-42,44-50]
Information systems and technologies	Using information systems and technologies to support routine clinical care, business processes, and patient-centered service provision	[2,9,30-35,37-39,41-48,50]
Patient focus	Patient empowerment, use of technology for self-management and wellness, patient eHealth literacy, and education	[2,9,31-33,35,37,39-43,46]
Health analytics	Use of data analytics in practice for informed decision making, quality improvement, service planning, and delivery	[2,9,31-33,37,39-44,46-47]

**Table 2:** Fundamental themes of eHealth capability identified from focus groups.

Fundamental themes	Subthemes
Reinforce fundamental capabilities	Quality and safety, Communication, problem solving, critical analysis, patient-centeredness, professionalism, lifelong learning skills
Acknowledge and adapt existing capabilities	Understand purpose of systems, advanced digital literacy, adaptive behavior, active participation in codesign
Introduce and provide opportunities for new learning	Working with health data, integration of health information sources, eHealth-enabled new models of care, data analytics, data governance, data privacy, data security, shifting role of the health care professional

**Table 3:** Overarching domains of eHealth capabilities.

Number and domain		Capability statement
1	Digital technologies, systems, and policies	Understand the purpose and function of digital health technologies and systems implemented at a local, state, or national level, including consideration of legal, policy, and ethical implications
2	Clinical practice and applications	Integrate digital health into clinical practice to deliver safe and quality care, including provision of best practice models of care
3	Data analysis and knowledge creation	Use data and data analysis to inform, deliver, and improve health and health care practice at an individual, team, or systems level
4	System and technology implementation	Participate in digital health implementation, evaluation, and codesign processes to drive improvement and stimulate change

## Discussion

### Principal Findings

This study aimed to develop a framework of eHealth capabilities that could be used to inform development of higher education curricula for health students and professional development opportunities for the current health workforce. The framework resulting from our literature review, focus groups, and Delphi process describes 4 overarching domains of eHealth capabilities: (1) digital technologies, systems, and policies; (2) clinical practice and applications; (3) data analysis and knowledge creation; and (4) system and technology implementation. Each domain is supported by a capability statement that articulates what learners need to be able to know and do to demonstrate achievement of this capability. Examples of these knowledge and behaviors are provided in the form of performance cues. The key components of the eHealth capability framework are provided in Multimedia Appendix 1.

The systematic process for developing the framework was necessary for the successful integration of eHealth and continuous quality improvement at all levels in the health system [9]. Critical to long-term implementation and adoption of the framework is the capability approach, which contrasts with a competency approach by recognizing the complexity and ever-changing nature of eHealth in practice. The idea of capability not only addresses complexity and evolving contexts but also incorporates the aspects of critical analysis and ethical practice in health care [25]. Furthermore, this research identified that eHealth capabilities should be integrated with clinical competencies and be sufficiently flexible so that they can be adapted to specific contexts.

Given the inherent nature of eHealth as being innovative and transformative, it is critical that we enable our health workforce to be suitably prepared and adaptable. Tertiary education of future health graduates needs to move from a focus on technical skills to encompass broader eHealth capabilities, such as professional competencies and attributes of an adaptable, improvement-minded, and innovative workforce. The eHealth capabilities framework extends beyond this technical proficiency to include the integration of technology into current practice and demonstrating a strong ethos around lifelong learning and transforming care. By re-evaluating traditional resources used in education and incorporating knowledge around eHealth capabilities, educational curriculum will provide greater opportunities to build capability in working with systems undergoing digital transformation [3]. Consistent with previous research [4,9], we identified the need for educational curriculum to reinforce existing eHealth capabilities, adapt existing capabilities to make them transferable to novel eHealth contexts, and introduce new learning opportunities for interactions with technologies within education and practice encounters. In essence, the capability framework developed assists in bridging the gap between academia and the application of digital health by emerging and existing health care professionals.

This framework is not a curriculum; it is a starting point to guide curriculum development and redesign. Furthermore, the framework is intended to stimulate discussion with industry stakeholders regarding workforce capability with regard to eHealth in practice. As a guide, it will enable further development of both curriculum and competency evaluation [48]. The eHealth capabilities framework is not intended to set a rigid curriculum for eHealth education, but rather to provide a key resource and common standards for the review, development, and alignment of profession-specific curricula to ensure high-quality and consistent student learning experiences. Attaining capability will ideally involve embedding eHealth within problem-based, case-based, and practice-based learning experiences and incorporate digital simulations and codesign projects. This work emphasizes core professional practice principles that underpin all activities involving eHealth, including quality and safety, consumer-centeredness, critical thinking, and evidence-based practice. As technologies evolve and practices involving eHealth grow, it is critical to maintain attention to these core principles. The inclusion of performance cues within the framework provides a starting point to describe how successful learning might be demonstrated. These are intended to guide the development of assessments that support the achievement of specific learning outcomes and activities of the discipline-specific curriculum.

It was clear that there is a need to create an eHealth capability framework that accurately reflected the roles and work contexts in the digital age. In addition to moving beyond a focus on the technical skills, this included offering theoretical and practical opportunities for health graduates to integrate eHealth into practice, eg, using technologies to provide new models of care and facilitate consumer empowerment, or to use routinely collected digital health data to inform practice. One of the key challenges for education in this space is the ability for curriculum to adequately address the divide between digitally capable individuals and their capacity to apply digital skills to clinical situations that often use archaic systems [49]. Although current students may now use digital technologies in their everyday lives, they might have limited knowledge of the opportunities and issues that technology can bring to the health care landscape [49]. As a result, students require tailored opportunities to ensure that they develop or translate skills and knowledge to effectively practice in evolving digital workplaces.

### Limitations

In this study, we did not aim to achieve data saturation as this was beyond the scope of this initial work. The participants in this study brought with them a breadth of experience and representation of multiple health professions, but the lack of consumer representation was a study limitation. It is likely that future research incorporating additional participants working across a wider range of contexts, and is inclusive of diverse health care consumers, may provide additional insights into eHealth-enabled interdisciplinary practice. Further research exploring the implications for the existing health care workforce is also warranted, with a focus on identifying the potential relevance and impact of the capability statements on policy and practice, including recruitment, professional development, performance management, and systems improvement activities. Building on the results of this study, the University is undertaking a curriculum mapping process with a commitment to the development of high-quality teaching and learning activities and resources. This will include an analysis of clinical placement experiences, work-based training, and workplace orientation programs to further identify and address gaps in the preparation of our health graduates and workforce for eHealth contexts. We further excluded from the discussion implications of automation and computerization on the actual workforce and their predicted displacement [50]. Thus, the impact artificial intelligence might have on the health care delivery system was not in the scope of this research.

### Practical Implications

This framework has direct implications for curriculum redevelopment in health education and professional development opportunities for the current health workforce. The framework could be used to assess which eHealth capabilities are currently being taught in health profession degrees, and how. Exemplars of effective eHealth education could be collated to form resources and professional development for health educators across the sector. Importantly, in using the framework to map current curricular, educators and course coordinators can identify the capabilities not addressed in their programs at present. This provides opportunity for revising the curriculum to better prepare graduates.

For the current health workforce, the framework requires further development. At present, the capabilities reflect the knowledge and skills required of a graduate. Further research and development are required to articulate intermediate and advanced levels of capability across the 4 domains of the framework. Once established, this more comprehensive framework could guide professional development opportunities and self-reflection or self-assessment for practicing health professionals.

### Conclusions

This paper describes the foundational level of eHealth capability expected of tertiary health students at graduation and as they enter the health workforce. Ideally, the eHealth capability framework will inform how tertiary health programs deliver and assess essential eHealth education. The results of this study will inform a cross-faculty eHealth curriculum that aligns with workforce expectations and will be of interest to professional associations, health services, and organizations. Future research needs to explore the potential for integration of findings into workforce development programs, particularly with consideration of intermediate and advanced levels of capability in collaboration with workforce and industry stakeholders.

## Appendix

Multimedia Appendix 1 eHealth capability framework.

## Abbreviations

eHealth: electronic health
