# Supplementary material for: An eHealth Capabilities Framework for Graduates and Health Professionals: Mixed-Methods Study
Source: J Med Internet Res. 2018 May 15;20(5):e10229. doi: 10.2196/10229 (PMC5974459; doi:10.2196/10229)
Supplement: Multimedia Appendix 1 [file jmir_v20i5e10229_app1.pdf]

## Multimedia Appendix 1. eHealth capability framework.

**Foundation Level for all Clinical Health Professionals**

This document is an outcome of the *eHealthMap*, a multi-faculty collaborative project at the University of Sydney, funded via an Education Innovation Grant (2016) from the Office of the Deputy Vice Chancellor (Education). The project was led by Tim Shaw, Professor of eHealth, Faculty of Health Sciences, and managed by the Research in Implementation Science and eHealth group. Special acknowledgement goes to the project Steering Committee and key project informants, which includes stakeholders from the Faculties of Health, Medicine, Nursing, Dentistry, and Arts and Social Sciences, as well as stakeholders from local, state, and national health districts; government instrumentalities and eHealth organizations. The project was developed in close association with the NSW Health Analytics Framework, Workforce Skills and Training Working Group chaired by Professor Annette Solman.

**Background**

Health service expectations for workforce-ready health professional graduates, competent to practice within digital health environments, has highlighted the need for universities to incorporate essential eHealth learning and practice experiences into degree programs. At the University of Sydney, the Health Faculties and Social Sciences have collaborated to develop eHealth capability statements to inform and guide consistent high-quality teaching and learning experiences. Digital systems and technologies increasingly impact on how we engage with regards to health and wellness. Today and into the future, all graduates of clinical health professional degree programs must be equipped with the requisite knowledge and skills to apply eHealth in their practice to collaborate, improve outcomes and transform care.

This document outlines foundation levels of knowledge and performance required by all healthcare professionals practicing in digital healthcare environments. This level directs the expectations of training and entry-level health professionals who provide direct clinical care to patients and who work under supervision across all sectors, that is, inclusive of all Australian health care settings.

**Underpinning principles for practice involving eHealth**

This work emphasizes core professional practice principles that underpin all activities involving eHealth, including quality and safety, person-centredness, critical thinking, and evidence-based practice. As technologies evolve and practices involving eHealth grow, it is critical to maintain

attention to these core principles. It is important that eHealth is considered in the context of delivering quality care rather than a series of new innovations that stand alone from practice.

**About the eHealth capability statements**

The eHealth capability statements have been built using the best available evidence from the literature and informed by the experiences and knowledge of healthcare workers, educators, and systems leaders. Statements align with capability expectations for entry-level clinical positions, therefore, levels of mastery are not specified. The capability statements are not intended to set a rigid curriculum for eHealth, rather, to provide a key resource and common standards for the review, development, and alignment of profession-specific curricula to ensure high-quality and consistent student learning experiences. Attaining competence will ideally involve embedding eHealth within problem-based, case-based and practice-based learning experiences, and incorporate digital simulations and co-design projects.

*Performance elements* provide a starting point to describe how successful learning might be demonstrated. These are intended to guide the development of learning outcomes or assessment criteria that best match with the specific learning outcomes and activities of the discipline specific curriculum adopted. Ideally, the capability statements will be integrated with the performance or competency-based assessment of the relevant professional competency-based occupational standards.

**Definitions of eHealth**

Within the Australian health context, the terms *eHealth* and *digital health* are used somewhat interchangeably with reference to the application of digital information and communication technologies and systems in the delivery of health services and practice of health and wellness. Definitions of eHealth are encompassing of, but not limited to, the use of digital technologies and systems to:

- Collect, manage, analyze, and apply digital health data. For example, electronic health record data, population health data, biobanks, patient-reported experiences and outcomes, consumer-generated data, administration and claims data.
- Monitor, track, and inform health and wellness. For example, mobile devices (e.g., smartphones, tablets, and clinical devices), mobile sensors and wearables, apps, social media, and online information;
- Facilitate communicative encounters between health stakeholders. For example, web-conferencing, SMS and push notifications from apps, patient storytelling through dedicated portals and social media platforms, and via virtual or simulated therapy tools; and

- Collect, manage, analyze, and apply digital health data. For example, electronic health record data, population health data, biobanks, patient-reported experiences and outcomes, consumer-generated data, administration and claims data.

**Assumed knowledge and skills**

It is assumed that all graduate healthcare professionals will have the following basic professional skills and therefore these are not specifically referenced in the capability statements:

- Basic ICT literacy
- Commitment to professional development and life-long learning
- Professionalism and ethical behaviour
- The ability to perform core duties associated with their role, including making sound clinical judgement with a person-centred focus, in the absence of digital systems or when digital systems are disrupted or fail.

| Capability Statement 1. Digital Technologies, Systems, and Policies |
|---|
| **LEARNING OBJECTIVE.** |
| **Understand the purpose and function of digital health technologies and systems implemented at local, state or national level, including consideration of legal, policy, and ethical implications.** |
| **KNOWLEDGE.** ***An understanding of:*** |
| 1. The key components of digital health systems at personal, local, state, and national levels within the Australian health context. For example, electronic health records and electronic medication systems.<br>2. The range, purpose and functions of various digital health technologies and systems used for the communication of health related information. For example, telehealth, secure messaging, web-based platforms, social media, apps, sensors, and monitors.<br>3. The fundamentals of digital health legislation, policy and ethics, including privacy and security, data governance, and professional conduct.<br>4. Governance of clinical information systems and the various stakeholders, positions, and privileges associated with the use of digital health technologies and systems.<br>5. The advantages and potential challenges of contemporary digital health technologies and systems, including the transience, interoperability, and limitations of technologies that are in place and the inherent risks that these may pose. |
| **PERFORMANCE ELEMENTS.** ***Demonstrates ability to:*** |
| 1. Be able to use computers and other devices competently to practice in a fully digital healthcare environment, including databases, word processing, apps, and online systems.<br>1. Access and navigate digital health technologies and systems relevant to scope of professional role.<br>2. Comply with national, state, and organizational legislation when using digital health technologies and systems.<br>3. Use digital health technologies and systems professionally and ethically while maintaining privacy and professional boundaries. For example, appropriate use of email, secure messaging, social media, and digital images. |

| Capability Statement 2. Clinical Practice and Applications |
|---|

| LEARNING OBJECTIVE. |
|---|
| **Integrate digital health into clinical practice to deliver safe and quality care, including provision of best practice models of care.** |
| **KNOWLEDGE.** ***A knowledge of:*** |
| 1. The impact of digital health on healthcare practice, patient safety, and the quality of care, including the potential for both positive and negative impacts on clinical practice and patient outcomes. For example, the impact of various digital health methods on service access, care coordination, and personalized and precision healthcare.<br>2. The impact of technology on communication within healthcare. For example, supporting digital or virtual teams.<br>3. The potential of digital health to support current practice and transform healthcare delivery by enabling new models of care. For example, the capacity of electronic health records, web-based consultations, and remote monitoring to enable integrated care, models of self-management, virtual care, and shared care.<br>4. The role of digital health in consumer engagement, including the impact of digital literacy and consumer-centred technologies and systems for health and wellness. For example, the impact of self-monitoring, digital information seeking, personally-controlled health records, consumer-centred apps, and patient portals.<br>5. The impact of digital health on the role of the health professional, including the changing dynamics between health care providers and consumers. For example, the roles of health professionals and consumers as partners and coaches in digital monitoring, data driven shared decision making, and social networking. |
| **PERFORMANCE ELEMENTS.** ***Demonstrates ability to:*** |
| 1. Use digital health appropriate to professional role, duties, and scope of practice in the provision of safe and quality care. For example, utilize electronic health records, medication systems, imaging systems, referral tools, and discharge summaries in the delivery of coordinated care.<br>1. Use digital health to support relationships and strategic communication with and between providers, teams, consumers, and the public. For example, utilize web-conferencing, secure messaging, and social media in networking, mentoring, consumer advocacy.<br>2. Access, aggregate, and cross-reference data from various sources, including those that do not readily interface, to generate a comprehensive clinical picture or summary of care, particularly at the point of transitions in care where system interoperability may not exist.<br>3. Engage in continuous critical appraisal of digital health and apply sound clinical decision making when technologies or systems are inappropriate, inaccurate, disrupted, or non-functional.<br>4. Maintain a consumer-centred approach to practice when using digital health. For example, consider consumer digital literacy levels, assess |

issues, preferences, and personal goals when using or prescribing digital health interventions.

5. Access and critically evaluate digital information, including appraisal of clinical data and health information within digital technologies and systems. For example, perform continuous appraisal of the quality and validity of digital health data contained within electronic health records, websites, and clinical decision support systems.
6. Critically evaluate digital health interventions, such as hardware, apps, software, web-based portals etc., to establish the evidence-base, as well as the benefits or risks to quality care and patient safety.
7. Create evidence-based digital health resources using digital information and multimedia. For example, use digital images and infographics for consumer information or personal and professional development.

| **Capability Statement 3**: **Data Analysis and Knowledge Creation** |
| --- |
| **LEARNING OBJECTIVE** |
| **Use data and data analysis to inform, deliver, and improve health and health care practice at individual, team, and systems levels.** |
| **KNOWLEDGE.** ***An understanding of:*** |

1. The data lifecycle, including the purpose and value of quality digital health data as it applies to evidence-based practice, decision making, value-based care, and quality improvement.
2. The range and purpose of data types and various databases, including linked data sets, electronic health record data, population health data, biobanks, patient-reported experiences and outcomes, consumer-generated data, and administration and claims data.
3. The potential of emerging digital data sources and data systems to transform health and healthcare. For example, the potential of big data, record linkage, data warehousing, and machine learning.
4. The range, purpose, and functions of basic data analytics tools in understanding changes in data over time in the clinical context. For example, the role of run charts in tracking performance, establishing intervention effectiveness, and informing quality improvement.
5. Data related terms and concepts, including metadata, risk adjustment, and source of truth.

**PERFORMANCE ELEMENTS.** ***Demonstrates ability to:***

1. Enter accurate, valid, and complete clinical data into digital health systems. For example, enter quality data into digital health records.
1. Access and apply various forms of digital health data relevant to professional practice, including consideration of new and emerging forms of data. For example, use biomedical information and patient-generated data in clinical decision making and delivery of personalised care.
2. Apply basic data analytics to unstructured digital data sets. For example, use basic analytics to identify trends, gaps, opportunities, and potential care trajectories for an individual or cohort of individuals, and generate basic data visualisations for progress tracking and outcomes reporting.
3. Apply basic interpretation, evaluation, and communication of statistical information.
4. Use digital data to monitor and reflect on personal performance and inform personal professional development.

**Capability Statement 4. System and Technology Implementation**

**LEARNING OBJECTIVE**

**Participate in digital health implementation, evaluation, and co-design processes to drive improvement and stimulate change.**

**KNOWLEDGE.** ***An understanding of:***

1. The basic processes involved in the implementation and evaluation of digital health technologies and systems.
2. The governance, personal-professional roles, and responsibilities in digital health implementations and evaluations within own work context.
3. The roles and responsibilities of the multiple stakeholders involved in the co-design and development of digital health technologies and systems, including considerations of system interoperability in transitions of care.
4. User adoption and change management issues and processes involved in incorporating technologies into practice. For example, an understanding of the health professionals' role as a champion or role model in enabling others to use digital technologies and being advocates for change.

**PERFORMANCE ELEMENTS. *Demonstrates ability to:***

1. Actively participate in the rollout of new digital health technologies and systems within own practice context.
1. Problem-solve and take actions to resolve basic technology challenges and disruptions that impact on professional practice and/or the delivery of safe and quality patient care.
2. Act in an improvement-minded way to affect change. For example, report digital health user experiences to relevant manager or department, present solutions, advocate for change, and participate in co-design processes as appropriate.
3. Remain up-to-date with digital health systems and technologies within own professional and work contexts.
